# Draft Genome Sequences of Three Bacterial Species from Aquatic Habitats near Washington, DC

**DOI:** 10.1128/mra.01319-22

**Published:** 2023-02-22

**Authors:** Riley T. Leff, Jonathan MacDougall, Christina Pavloudi, Lausanne Oliver, Kaitlynn Slattery, Guinevere Lissner, Jimmy H. Saw

**Affiliations:** a Department of Biological Sciences, The George Washington University, Washington, DC, USA; University of Delaware College of Engineering

## Abstract

We report the draft genome sequences of three bacterial species isolated from freshwater ponds or features around monuments in Washington, DC, during a semester-long microbiology lab course at the George Washington University. Two of the isolates belong to potentially novel species but lost their viability and could not be revived.

## ANNOUNCEMENT

Three bacterial strains were isolated from aquatic habitats around Washington, DC, during a microbiology laboratory course (BISC2337) conducted at the George Washington University (GW) in the fall of 2021. Water samples from the Lincoln Memorial Reflecting Pool, Constitution Gardens Pond, and Tidal Basin were collected by course instructors and some of the undergraduate students enrolled in the course (Table 1). Wearing nitrile gloves, water samples were collected into sterile 50-mL centrifuge tubes, kept in Ziploc bags, transported back to GW on foot, and kept in a 4°C fridge. Undiluted water samples were spread-plated on nutrient agar (NA) (RPI; N14100-500.0) and incubated at 30°C, and the isolated colonies were streaked again on nutrient agar plates to obtain pure cultures. Colony PCR was performed as previously described (1, 2) on single isolated colonies using the primers 515F (GTGCCAGCMGCCGCGGTAA) and 1492R (TACGGYTACCTTGTTACGACTT) to amplify their 16S rRNA gene. Sanger sequencing was performed on the purified PCR products (Qiagen QIAquick PCR purification kit). Based on the best BLAST matches against the NCBI nonredundant/nucleotide (nr/nt) database, some isolates were selected for shotgun genome sequencing.

**TABLE 1:** List of bacterial isolates, assembly statistics, and basic metadata

Parameter	Data for strain:		
	*Erythrobacter* sp. WG	*Niveibacterium* sp. 24ML	Pseudomonas sp. TB1-B1
Class	*Alphaproteobacteria*	*Betaproteobacteria*	*Gammaproteobacteria*
Closest type strain relative^*a*^	Erythrobacter colymbi JCM 18338	Niveibacterium umoris DSM 106739	Pseudomonas asgharzadehiana SWRI132
Best-matched organism^*b*^	*Porphyrobacter* sp. HT-58-2	Niveibacterium microcysteis HC41	Pseudomonas asgharzadehiana SWRI132
% identity	99.39	99.48	100
GenBank accession no.	CP022600.1	CP071060.1	CP077079.1
FastANI score (%)	83.28	82.27	99.57
Source of isolation	Lincoln Memorial Reflecting Pool	Constitution Gardens Pond	Tidal Basin
Cultivation medium	Nutrient agar	Nutrient agar	Nutrient agar
Growth temp (°C)	30	30	30
Assembly size (Mbp)	3.31	4.64	6.18
No. of contigs	58	69	42
Sequencing depth (×)	184	218	136
*N*_50_ (bp)	91,118	152,958	324,987
Completeness (%)	99.25	99.01	99.93
Contamination (%)	0.46	1.45	0.34
G+C content (%)	68.02	63.65	60.71
BioProject accession no.	PRJNA888806	PRJNA888806	PRJNA888806
BioSample accession no.	SAMN31221292	SAMN31221294	SAMN31221297
SRA accession no.	SRR21878372	SRR21878374	SRR21878377
WGS^*c*^ assembly accession no.	JAPFGY000000000	JAPFHA000000000	JAPFHD000000000

a Based on TYGS analysis.

b Based on predicted 16S rRNA gene sequence.

c WGS, whole-genome sequencing.

The isolates were grown on nutrient agar plates for several days. Genomic DNA was extracted from isolate WG and 24ML cells using the ZymoBIOMICS DNA miniprep extraction kit, while genomic DNA was extracted from isolate TB1-B1 using the NucleoSpin tissue XS kit. The DNA was sent to SeqCenter (Pittsburgh, PA), where library preparation and sequencing were performed by the company. Sequencing libraries were prepared using the Illumina DNA prep kit and IDT 10-bp unique dual indexes (UDI) and sequenced on an Illumina NextSeq 2000 instrument, producing 2 × 151-bp reads. Default parameters were used for all software unless otherwise specified. Demultiplexing, quality control, and adapter trimming were performed using BCL Convert v3.9.3 (3). The raw Illumina sequences were trimmed using BBDuk v38.21 (https://sourceforge.net/projects/bbmap/) (4) and assembled using SPAdes v3.13.0 (5). Assembly metrics were determined using QUAST v5.0.2 (6). Assessment of genome completeness and contamination was performed using CheckM v1.1.0 (7). Contigs larger than 1,000 bp were annotated using Prokka v1.14.5 (8). The draft genome assemblies were also submitted to NCBI and annotated by the NCBI Prokaryotic Genome Annotation Pipeline (PGAP) v6.3 (9). The sequencing depth was calculated using the Seqtk tool (https://github.com/lh3/seqtk) to calculate the total number of raw untrimmed bases and divide it by the genome assembly size of contigs larger than 1,000 bp.

Comparison of the draft genome sequences with type strains using the Type Strain Genome Server (TYGS) (10) and FastANI (11) to search against the genomes of closest relatives revealed that two of them (isolates WG and 24ML) likely belong to previously uncharacterized species, based on the Genome-to-Genome Distance Calculator results (https://doi.org/10.6084/m9.figshare.21675224.v1) and average nucleotide identity scores (Table 1). However, despite our best efforts to maintain the pure cultures, isolates WG and 24ML stopped growing after several rounds of restreaking on nutrient agar. We hope that the draft genome sequences of these uncharacterized bacteria may be useful for comparative genomic analyses and for predicting their metabolic requirements to design a medium better suited for their growth.

### Data availability.

The NCBI BioProject accession number for this project is PRJNA888806. The raw Illumina reads have been deposited in NCBI Sequence Read Archive (SRA) under the following accession numbers: SRR21878372, SRR21878374, and SRR21878377. The whole-genome sequence (WGS) assemblies have been deposited at NCBI GenBank under the following accession numbers: JAPFGY000000000, JAPFHA000000000, and JAPFHD000000000.
